# Perceived negative consequences of dyslexia: the influence of person and environmental factors

**DOI:** 10.1007/s11881-022-00274-0

**Published:** 2022-11-30

**Authors:** Loes Bazen, Elise H. de Bree, Madelon van den Boer, Peter F. de Jong

**Affiliations:** 1 Research Institute of Child Development and Education, University of Amsterdam, P.O. box 15780, 1001 NG Amsterdam, the Netherlands; 2 Present Address: Department of Education and Pedagogy, Utrecht University, P.O. Box 80140, 3580 TC Utrecht, the Netherlands

**Keywords:** Dyslexia, Environmental factors, Perceived consequences, Person factors

## Abstract

Perceived negative consequences of dyslexia entail the degree to which an individual perceives negative outcomes, such as low academic achievement or feelings of anxiety and depression, and attributes these experiences to the disorder. In the current study, we examined how perceived consequences of dyslexia are influenced by person and environmental factors. Perceived consequences were evaluated for the academic domain and the domain of mental health (depression, anxiety). Participants were 123 Dutch students with dyslexia. Cognitive person factors (literacy skills and verbal IQ), socio-emotional person factors (self-perceived literacy skills and coping ability), and environmental factors (literacy demands, support from the institution, reactions of teachers and peers) were included as predictors. Results indicated that perceived negative consequences were not related to cognitive person factors. In contrast, better self-perceived literacy skills were associated with less perceived negative consequences in all domains (academic, depression, anxiety) and coping contributed to depression consequences. With respect to environmental factors, negative reactions in the academic environment contributed to perceived negative consequences of depression and anxiety. As such, findings indicate that individuals with dyslexia perceive negative consequences in the academic, anxiety, and depression domains which cannot be fully accounted for by their objective reading and writing problems. These factors should feature more prominently in future studies on dyslexia and should be addressed in treatment of dyslexia as well.

**Supplementary Information:**

The online version contains supplementary material available at 10.1007/s11881-022-00274-0.

Dyslexia is characterized by severe and persistent difficulties with word reading and/or poor spelling (e.g., Lyon et al., 2003). These difficulties can have negative effects on educational attainment (Richardson & Wydell, 2003; Stampoltzis & Polychronopoulou, 2009). In addition, dyslexia is associated with internalizing problems such as anxiety and depression (meta-analyses by Francis et al., 2019; Gibby-Leversuch et al., 2019). Despite these known negative effects at the group level, people with dyslexia tend to differ in the degree to which they believe that their literacy difficulties cause problems in academic achievement and feelings of anxiety and depression. Some people with dyslexia perceive the consequences of their dyslexia as very severe, whereas others hardly seem to experience any burden of the disorder (e.g., Burden, 2008; Ingesson, 2007; Ruijssenaars et al., 2008). A distinction can thus be made between the actual consequences of the disorder and its perceived (negative) consequences, that is the extent to which negative experiences are believed to be caused by (attributed to) the disorder. In the present study, we examined the factors that influence the extent to which university students with dyslexia believe their disorder to impede academic performance and cause feelings of anxiety and depression.

A general model for understanding the (perceived) consequences of a disorder is the International Classification of Functioning, Disability and Health (ICF; WHO, 2001). The model facilitates the identification of specific factors within a person’s life that may affect the degree to which they experience consequences from the disorder. In the model, two categories of factors are distinguished that can influence functioning in terms of activities and participation of a person with a disability. These are person factors, referring to internal influences, and environmental factors, referring to external influences on functioning. Translating this model to the context of university students with dyslexia, person factors entail both cognitive person factors, including the severity of the literacy disorder, and socio-emotional factors. Environmental factors could refer to support provided to the person with dyslexia. Factors in the ICF model can influence the perceived consequences of the disorder either positively or negatively. This implies that this perception is believed to be determined by the severity of the disorder itself, as well as by other person and environmental factors (see, for example, Sims et al., 2021).

Multifactorial models that concern risk and protective factors in the development of dyslexia itself have also stressed the role of person and environmental factors (e.g., Catts & Petscher, 2022). So far however, not much attention has been paid to the specific factors that influence perceived consequences from dyslexia in the academic domain and the domain of mental health. As an exception, Elbro (2010) evaluated the influence of cognitive person factors on this perception. Individuals with dyslexia of various ages and educational backgrounds were asked about the academic or broader study-related consequences they experienced from their dyslexia. In addition, literacy ability, phoneme awareness, and general verbal ability (vocabulary) were assessed. Elbro found that individuals with higher vocabulary were more likely to experience negative academic consequences from their dyslexia than individuals with lower vocabulary after word reading ability was controlled for. Hence, for this group, higher general verbal ability was related to more perceived negative academic consequences. Elbro suggested that individuals with higher general verbal ability could experience more negative consequences because they might set goals involving high literacy demands. In line with the ICF model, Elbro’s study thus shows that factors next to or above the severity of the disability contribute to the extent to which negative consequences of the disorder are perceived.

Next to cognitive person factors, the ICF model indicates that socio-emotional person factors may contribute to the experience of negative consequences from dyslexia. One such factor is self-perceived literacy (dis)ability. This sub-component of academic self-concept (Marsh, 1990) might be low(er) in persons with dyslexia, as their literacy ability leads to negative experiences in reading and spelling. Meta-analyses show that dyslexia is not related to global self-concept or self-esteem, but that persons with dyslexia do have a lower self-concept in the areas that are related to literacy (Gibby-Leversuch et al., 2019; McArthur et al., 2020). Other studies show that the relations between domain-specific self-concept and academic achievement are reciprocal (Marsh & Craven, 2006; Marsh & O’Mara, 2008). Similarly, there might be a reciprocal relation between self-perceived literacy ability and the perceived academic consequences of dyslexia.

Self-perceived literacy ability may also be related to perceived negative consequences from dyslexia in the domain of mental health. In general, relations between overall academic self-concept and internalizing problems have been reported. A higher academic self-concept has, for example, been associated with less test-anxiety (Zeidner & Schleyer, 1999). Moreover, low general self-esteem has been shown to be a risk factor for depression (Baumeister et al., 2003; Sowislo & Orth, 2013). More generally, Burden (2008) found that the poor overall academic self-concept of people with dyslexia was related to less ability and motivation to learn, because literacy problems reduced the confidence to succeed. Relations between academic self-concept and internalizing problems (anxiety and depression) yield the inference that self-perceived literacy, a narrower notion of self-concept, is related both to feelings of anxiety and depression.

Another socio-emotional person factor that could affect the negative consequences students perceive from dyslexia is coping, referring to ‘the behaviours a person uses to meet his[/her] own needs and to adapt to the needs of the environment’ (Fine et al., 1984, cited by Cowen, 1988, p 161). For students with dyslexia, coping mechanisms related to learning (problem-focused coping, see Baker & Berenbaum, 2007) might entail knowing how to deal with dyslexia in an academic environment, for example, by making use of appropriate learning strategies. Alternative coping strategies, such as task avoidance (emotion-focused coping), may lead to negative feelings and stress (Pirttimaa et al., 2015). Individuals with dyslexia have been found to differ in the quality of their coping strategies (Stampoltzis & Polychronopoulou, 2009). Problem-focused coping among individuals with dyslexia has been associated with factors that are beneficial for learning, such as motivation to persist in learning activities (Singer, 2008), pro-active behavior (Alexander-Passe, 2006), and internal locus of control (Firth et al., 2013). Also, successfully employing coping strategies has been associated with fewer feelings of depression in individuals with dyslexia (Alexander-Passe, 2006). Although the exact impact of coping as a factor that influences the perceived consequences from dyslexia is unknown, these relations between coping strategies and academic success as well as emotional well-being indicate that successful coping may diminish perceived negative consequences in the academic domain and the domain of mental health, in particular with respect to anxiety and depression.

Following the ICF model, also environmental factors are expected to influence the perceived consequences of dyslexia. An academic context, for example, sets high literacy demands and may increase the negative academic consequences that people with dyslexia experience. Indeed, an interview study by Pirttimaa et al. (2015) established that the high literacy demands that students with dyslexia in higher education encountered, such as having to read long texts, writing essays, and using foreign languages, impacted strongly on their experienced academic success. High literacy demands may aggravate the perception of negative academic consequences of dyslexia, although quantitative research supporting this claim is lacking.

Although not investigated in the light of perceived consequences, some studies also point towards a relation between literacy demands and internalizing problems. High school students with dyslexia were found to have higher levels of anxiety when reading than their peers (Carroll & Iles, 2006). In addition, the stress that comes with generally high university demands has been associated with feelings of depression among university students in general (Dahlin et al., 2005). Individuals with dyslexia may therefore experience feelings of anxiety and depression that are due to their literacy problems when demands are high. High literacy demands may thus contribute to perceived negative consequences of dyslexia in these domains.

Other environmental factors that potentially affect the perceived consequences of dyslexia are related to educational support. It can be assumed, for instance, that more facilities provided by the educational institute decrease the perception of negative academic consequences of dyslexia. Such facilities may include accommodations to minimize the effects of dyslexia during exams (Lai & Berkeley, 2012), such as extended time (Bolt et al., 2011), leniency towards spelling errors, and feedback concerning these errors on tests and other work. Gibson (2012) showed that lack of support can also have an emotional impact and may consequently influence perceived consequences related to feelings of anxiety and depression. Thus, although research has not examined the effect of educational support on perceived consequences of dyslexia themselves, it seems likely that the amount and quality of support of the institution is related to the perception of these consequences among students with dyslexia.

Attitudes of lecturers and peers also form an environmental factor within the educational institution: positive attitudes of lecturers and peers might reduce experienced negative consequences of dyslexia. Research has shown the importance of lecturers’ attitudes for well-being and successful learning (Roorda et al., 2011), especially for individuals with dyslexia (Burden & Burdett, 2005; Glazzard, 2010; Humphrey, 2003; Humphrey & Mullins, 2002). Furthermore, Nielsen (2011) reported that for individuals with dyslexia of various ages, support of teachers and their understanding was appreciated more strongly than good teaching methods. However, emotional support, closeness, and understanding are not always experienced by elementary students with dyslexia (Humphrey & Mullins, 2002; Zee et al., 2020). The importance of lecturers’ support has also been shown for students in higher education with disabilities, including dyslexia (Gibson, 2012). The quality of perceived support of teachers and lecturers may therefore contribute to perceived academic consequences and consequences in the domain of internalizing problems for this group as well.

There are comparable findings with respect to peer support in relation to academic success and emotional well-being. Younger students with dyslexia have reported being teased and bullied by peers as well as feeling excluded (Glazzard, 2010; Humphrey, 2003; Humphrey & Mullins, 2002; Morgan et al., 2012). At the same time, peers can also contribute to positive emotions and a good learning climate when they provide security and aid in revising and preparing assessed work (Gibson, 2012; Gibson & Kendall, 2010; Humphrey, 2003). Although Gibson (2012) found that mature and supportive attitudes from peers were experienced by students with dyslexia at university, bullying is still experienced (Morris & Turnbull, 2006). Given the associations between good peer relations and emotional well-being as well as academic support, it can be expected that fewer negative consequences of dyslexia in all domains are experienced by students with more positive peer relations.

## Current study

**Fig. 1:**
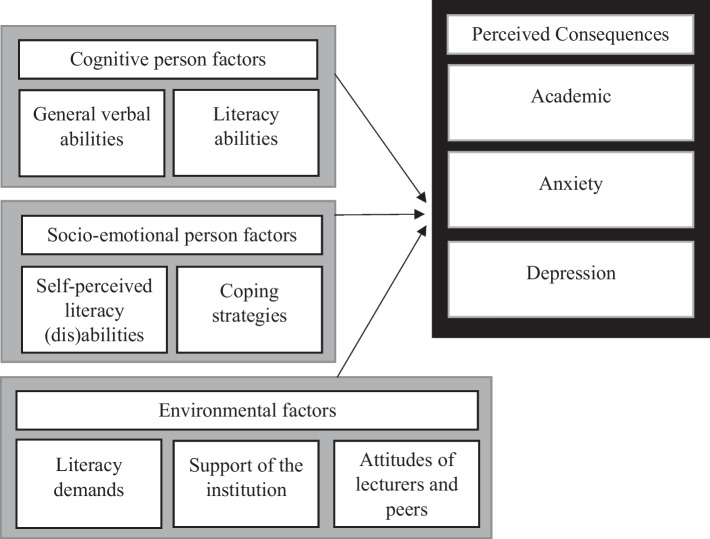
Schematic summary of potential factors of influence on perceived negative consequences of dyslexia

The literature has provided insights into academic attainment, anxiety, and depression as secondary consequences of dyslexia, as well as into relations between these consequences and internal and external factors. Yet, little is known about the relation between these factors and *perceived* consequences of the disorder. In the present study, we investigated how such experienced negative consequences in the academic domain and the domain of mental health (anxiety and depression) among Dutch university students with diagnosed dyslexia were related to cognitive and socio-emotional person factors as well as to environmental factors (summarized in Fig. 1). Cognitive person factors included literacy ability and verbal intelligence. Socio-emotional person factors included self-perceived literacy problems and coping. Environmental factors included literacy demands, support of the institution, and attitudes of lecturers and peers.

To our knowledge, only one previous study by Elbro (2010) has focused on factors that are associated with the perceived negative consequences from dyslexia. On the basis of this study, we expected that severity of the literacy disability would contribute to the extent that negative consequences are attributed to dyslexia in all domains and that higher verbal ability would lead to more severe perceived academic consequences.

As for the relation of other person and environmental factors related to perceived negative consequences, predictions were less clear-cut. It follows from the ICF model (WHO, 2001) that in addition to cognitive factors, also socio-emotional person and environmental factors will be related to the perceived consequences of dyslexia. But there is a lack of quantitative empirical studies about the person and environmental factors associated with the experienced consequences in various domains. Yet, from the mostly qualitative studies mentioned above, it might be hypothesized that both positive person factors (larger self-perceived literacy ability and better coping) and more favorable environmental factors (lower literacy demands, institutional support, positive attitudes of peers and teachers) are related to less perceived consequences of dyslexia in both the academic domain and the domain of mental health.

## Method

### Participants

Participants were Dutch students who had been formally diagnosed with dyslexia. This diagnosis had always been established by a certified healthcare professional. Following international criteria of the DSM-5 (American Psychiatric Association, 2013), criteria for a diagnosis in the Netherlands entail persistent and severe word-level reading and/or spelling difficulties (< 10th percentile; SDN, de Jong et al., 2016). This means that, despite adequate instruction and learning abilities, word-level literacy problems do not decrease. IQ is not part of the exclusion criteria for a dyslexia diagnosis (except in the case of established general learning difficulties), but sensory deficits or limited opportunity to learn were.

The primary sample consisted of 131 students. Participants older than 30 years were excluded (*n* = 6). Furthermore, data was excluded from two students who did not complete all of our tasks. The final sample consisted of data of 123 Dutch university students with dyslexia (75% women, *n* = 92) with a mean age of 23.0 years (age range 18.9–30.4 years, *SD* = 2.5 years). The majority (*n* = 95) were recruited through calls on websites of educational institutions, social media, personal network, flyers, and posters. The other 28 students had already completed the cognitive tasks of the current study during a dyslexia examination procedure and completed the remaining part of the study.

All participants attended universities (*n* = 56, 46%) or universities of applied science (*n* = 63, 51%) at the time of testing, or had graduated within the past 6 months. This information was missing for four participants. Most students were enrolled in or had graduated from a study program in humanities, arts, social, or behavioral sciences (*n* = 69, 56%) and others in the area of science, technology, engineering, or mathematics (*n* = 32, 26%), or in other or combined areas (*n* = 16, 13%). This information was missing for six participants. The imbalance between male and female participants in the current study was probably caused by the higher number of female students at the specific educational institutions where students were recruited, as well as by the personal network of the female student-assistants who helped during the recruitment procedure.

All universities have to provide support to students with dyslexia and have policies in place to do so; see for example https://student.uva.nl/en/topics/studying-with-a-disability-dyslexia-or-chronic-illness and https://student.uva.nl/en/topics/studying-with-a-disability-dyslexia-or-chronic-illness for information on the policies of the (applied) universities. They are also guided by general principles of the expertise center on inclusive (tertiary) education (https://ecio.nl/en/). Generally, support refers to extended time during exams, font sizes of exams, support provided by the student counsellor, and specific training sessions for studying with dyslexia.

## Measures

### Cognitive person factors

#### Word reading fluency

Word reading fluency was assessed with the *One minute test*, version A (Een Minuut Taak; Brus & Voeten, 1999). The task consisted of 116 words of increasing difficulty that had to be read as quickly and accurately as possible in the time span of one minute. The score consisted of the number of words read correctly. Test–retest reliability is reported to be between 0.89 and 0.92 (Brus & Voeten, 1999).

#### Pseudoword reading fluency

Pseudoword reading fluency was assessed with the *Klepel*, version A (Van den Bos et al., 1994). The task consisted of 116 pseudowords of increasing difficulty that had to be read as quickly and accurately as possible in the time span of two minutes. The score consisted of the number of pseudowords read correctly. Reported test–retest reliability is 0.91 (van den Bos et al., 1994).

#### Word-level spelling

Spelling was assessed with the word dictation task from the *Test for Advanced Reading and Writing* (Test voor Gevorderd Lezen en Schrijven*,* Depessemier & Andries, 2009). This task consisted of 40 words (e.g., *dorpsschool* [village school]) and 10 pseudowords (e.g., *enkleun*) that were dictated one by one without time pressure. All words were repeated at the end of the test. Scores consisted of the total number of correctly spelled items, with a maximum score of 50. Split-half reliability is between 0.69 and 0.80 (Depessemier & Andries, 2009).

#### Verbal intelligence

To assess verbal intelligence, the Dutch version of the *Similarities* subtest of the *Wechsler Adult Intelligence Scale* (WAIS; Wechsler, 1981) was used. This subtest consisted of 19 items of increasing difficulty. For each item, participants were asked to name the similarity between two words (e.g., “car” and “airplane”). For items 1 to 5, one point was awarded per correct answer. For items 6 to 19, one point was assigned for functional answers (e.g., “car and airplane both need gasoline”) and two points for higher order answers (e.g., “car and airplane are both means of transportation”). The maximum score was 33. The reported test–retest reliability of the subscale is 0.91 (Brown & May, 1979).

### Perceived consequences, socio-emotional person factors, and environmental factors

We developed a questionnaire to measure the three forms of perceived consequences and the socio-emotional person and environmental factors. The questionnaire consisted of 70 items in total, divided in eight subscales. Three subscales concerned the three perceived consequence types: perceived academic, anxiety, and depression consequences. Two subscales concerned the socio-emotional person factors: self-perceived literacy disability and coping. Three subscales concerned the environmental factors: literacy demands, support from the educational institution, and attitudes of lecturers and peers. The items for the scales were formulated on the basis of literature on the specific topics and two in-depth interviews with students with dyslexia about these topics.

For each scale, a principal component analysis was conducted with the number of components to extract set to 1. Only items with factor loadings higher than 0.50 in the component matrix were retained. Subsequently, a reliability analysis was performed for each scale, in which items with an item-total correlation smaller than 0.30 were removed. This resulted in the removal of 19 items of the questionnaire, ranging from zero to five items per scale. Scales are described below. All included items can be found in the Supplementary Material.

#### Perceived academic consequences

The subscale *perceived academic consequences* consisted of seven items (based on Elbro, 2010). All questions were answered on a 5-point Likert scale (1 = “does not fit at all” to 5 = “fits well”). An example is: “If I did not have dyslexia, I would write better essays or papers.” The score consisted of the mean score for all items in the scale. Cronbach’s alpha for this subscale was 0.82.

#### Perceived anxiety consequences

The subscale *perceived anxiety consequences* consisted of five items (based on the Profile of Mood States questionnaire (McNair et al., 1989)). Questions were answered on a 5-point Likert scale (1 = “never” and 5 = “(almost) always”). An example of an item is: “Because of my dyslexia, I feel nervous.” The score consisted of the mean score for all items in the scale. Cronbach’s alpha was 0.71.

#### Perceived depression consequences

The subscale *perceived depression consequences* consisted of nine items (based on the Profile of Mood States questionnaire (McNair et al., 1971), and Humphrey & Mullins, 2002). Questions were answered on a 5-point Likert scale (1 = “never” and 5 = “(almost) always”). An example is: “Because of my dyslexia, I feel sad.” The score consisted of the mean score for all items in the scale. Cronbach’s alpha was 0.93.

#### Self-perceived literacy disability

The subscale *self-perceived literacy disability* consisted of nine items of the Reading Ability in Dutch Questionnaire (Van Bergen & De Jong, unpublished). Questions were answered on a 5-point Likert scale (1 = “never” and 5 = “(almost) always,” with one item on a different 5-point Likert scale: 1 = “very fast reader” and 5 = “very slow reader”). An example is: “I read more slowly than my study peers.” The score consisted of the mean score for all items in the scale. Cronbach’s alpha was 0.76.

#### Coping

The subscale *coping* consisted of five items (based on Stampoltzis & Polychronopoulou, 2009) that were answered using a 5-point Likert scale (1 = “does not fit at all” to 5 = “fits well”). An example of an item is: “I know how to deal with my dyslexia.” The score consisted of the mean score for all items in the scale. Cronbach’s alpha was 0.82.

#### Literacy demands

The subscale *literacy demands* consisted of six items based on the two interviews conducted in light of the current study that were answered on a 5-point Likert scale (1 = “never” and 5 = “(almost) always”). An example is: “Exams contain a lot of text.” The score consisted of the mean score for all items in the scale. Cronbach’s alpha was 0.82.

#### Support of the educational institution

The subscale *support of the educational institution* consisted of three items (Bolt et al., 2011). All questions were answered on a 5-point Likert scale (1 = “strongly disagree” to 5 = “strongly agree”). An example of an item was: “I am satisfied with the accommodations that my education institution offers.” These accommodations referred to those offered by the (applied) university the students were attending. The score consisted of the mean score for all items in the scale. Cronbach’s alpha was 0.82.

#### Attitudes of lecturers and peers

The subscale *attitudes of lecturers and peers* consisted of seven items (based on Gibson (2012), and Nielsen (2011)). All questions were answered on a 5-point Likert scale (1 = “does not fit at all” to 5 = “fits well”). An example is: “Lecturers take the time if I need more support due to my dyslexia.” Four items were formulated negatively (e.g., “Lecturers question my needs for support”) and those scores were reversed. The score consisted of the mean score for all items in the scale. Cronbach’s alpha was 0.73.

### Procedure

The students who were recruited for this study (*n* = 95) completed the literacy ability and verbal intelligence tasks followed by a questionnaire during an individual session with a trained student assistant. Testing took place in a quiet room within the university of the participant. On average, completing tasks and filling out the questionnaire (pen and paper) took around 60 min.

Students who had previously completed literacy ability and verbal intelligence tasks for diagnostic assessment (*n* = 28) were invited via email to complete the questionnaire online using Qualtrics software. Filling out the online questionnaire took 30 min on average.

All participating students received a gift voucher as compensation for their time and effort. Prior to data collection, approval was obtained from the Ethics Review Board of the Department of Social and Behavioral Sciences of the  University of Amsterdam (project number  2016-CDE-7457).

## Results

### Data screening and descriptive statistics

Before running our analyses, data was checked for outliers. The score of one (male) participant on the depression scale was considered an outlier (more than three standard deviations above the mean). We kept this score in the dataset, as removal did not change the results. There were no other outliers. All variables were normally distributed.

Descriptive statistics of all variables are presented in Table 1. Ranges and standard deviations for perceived negative consequences in all three domains indicated there was variability among scores. Numerically, perceived academic (3.32 out of 5) and anxiety consequences (2.95 out of 5) showed higher means than perceived depression consequences (1.97 out of 5). The ranges and standard deviations of cognitive, socio-emotional, and environmental factors also showed substantial variability and comparable total mean scores.

**Table 1:** Descriptive statistics

Scale	*M*	*SD*	Range
Perceived negative consequences			
Academic	3.32	0.78	1.25–5.00
Anxiety	2.95	0.80	1.00–5.00
Depression	1.97	0.81	1.00–4.33
Cognitive person factors			
Word reading fluency	76.24	13.62	45–106
Pseudoword reading fluency	68.04	17.36	19–106
Spelling	32.76	4.76	21–43
Verbal intelligence	24.72	3.62	16–32
Socio-emotional person factors			
Self-perceived literacy disability	3.72	0.58	1.89–5.00
Coping strategies	3.43	0.80	1.20–5.00
Environmental factors			
Literacy demands	3.35	0.72	1.33–5.00
Support institution	3.38	0.98	1.00–5.00
Attitudes of lecturers and peers	3.46	0.67	1.29–4.71

### Preliminary analyses: controlling for sex

Studies indicate that women tend to experience more internalizing problems than men (e.g., Altemus et al., 2014; McLean et al., 2011). Women may also be more likely to attribute such problems to dyslexia. In addition, relations between the person and environmental factors and the perceived consequences of dyslexia might differ between the groups. Such differences would imply that data of man and woman should be analyzed separately.

To test for these differences, regression analyses were performed for each combination of the 9 person and environmental factors with the three perceived consequences. For each of these 27 stepwise regression analyses, sex and one of the independent variables were entered in step one, followed by the interaction of sex and the variable of concern in step two. A significant interaction would indicate that the relation between the particular person or environmental factor and a particular perceived consequence would differ between men and women.

We observed only one significant interaction effect, between support of the institution and sex (β = 0.965, *p* = 0.021). Because only one out of 27 interactions turned out to be significant, this one interaction effect might have occurred by chance. Overall, the relations of person and environmental factors with perceived consequences were similar for men and women, and further analyses were performed on the full sample. Note that when we did add the main effect of sex in our regression analyses, this did not affect the pattern of results either.

### Correlations among person and environmental factors and perceived consequences

Correlations were computed among the three perceived negative consequences (academic, anxiety, and depression) and the person and environment factors. Also correlations of the person and environmental factors with the perceived consequences were computed. There were moderate positive correlations among the three types of perceived consequences (see for the qualification of the strength of a correlation, Cohen (1988)). Perceiving more academic consequences was related to more perceived anxiety consequences (*r* = 0.429, *p* < 0.01), perceiving more academic consequences was related to more perceived depression consequences (*r* = 0.540, *p* < 0.01), and perceiving more anxiety consequences was related to more perceived depression consequences (*r* = 0.545, *p* < 0.01).

Correlations among the person and environmental factors are presented in Table 2. Most factors did not correlate or correlated weakly. However, the correlation between the reading measures (word reading fluency, pseudoword reading fluency) was strong, and the correlation between spelling and pseudoword reading fluency was moderate. Notably, verbal intelligence correlated with none of the other variables. Self-perceived literacy disability was weakly related to word reading fluency and moderately to pseudoword reading fluency, but not to spelling. Moderate correlations were found between the two measures tapping into environmental support (support of institution, attitudes of lecturers and peers), as well as between these two factors and literacy demands. Generally, correlations indicated that the variables were largely independent, most clearly for socio-emotional person and environmental factors.

**Table 2:** Correlations among person and environmental factors

Factors	1	2	3	4	5	6	7	8
1. Word reading fluency								
2. Pseudoword reading fluency	.640**							
3. Spelling	.142	.344**						
4. Verbal intelligence	.011	.020	.134					
5. Self-perceived disability	− .211*	− .279**	− .162	.083				
6. Coping	− .040	− .073	.219*	.073	− .217*			
7. Literacy demands	− .200*	− .180*	.029	.071	.221*	-.010		
8. Support institution	.178*	.117	− .186*	− .024	− .127	.102	-.252**	
9. Attitudes of lecturers and peers	.064	.015	− .114	− .073	− .034	.000	-.346**	-.378**

^* ^*p* < .05. ** *p* < .01

The correlations of the person and environmental factors with the three types of perceived negative consequences are presented in Table 3. Generally, cognitive person factors did not correlate with the perceived consequences in all three domains. Verbal intelligence and spelling skills were not related to any of the perceived consequences. Lower word reading fluency and pseudoword reading fluency were weakly associated with more perceived consequences with respect to depression and anxiety, but were unrelated to perceived academic consequences.

**Table 3:** Correlations of person and environmental factors with perceived negative consequences

	Academic	Anxiety	Depression
Word reading fluency	− .059	− .195*	− .219*
Pseudoword reading fluency	− .074	− .192*	− .208*
Spelling	.014	− .061	− .022
Verbal intelligence	− .098	− .011	.039
Self-perceived literacy disability	.366**	.405**	.358**
Coping strategies	− .266**	− .087	− .259**
Literacy demands	.300**	.316**	.364**
Support institution	− .324**	− .263**	− .340**
Attitudes of lecturers and peers	− .231**	− .372**	− .350**

^*^ *p* < .05. ** *p* < .01

The socio-emotional person factor self-perceived literacy disability was positively correlated with all three types of perceived negative consequences (all moderate correlations). For the socio-emotional person factor coping, better coping was related to less perceived academic and depression consequences (weak correlations), but was not associated with perceived anxiety consequences.

The environmental factors all showed weak to moderate correlations with the three types of perceived consequences: lower literacy demands, better support of the institution, and better attitudes of lecturers and peers were all related to less perceived negative academic, anxiety, and depression consequences.

### Independent relations of person and environmental factors with perceived consequences

Next, we examined the independent or unique contribution of each factor to each of the three types of perceived negative consequences. We ran three multiple regression analyses, and therefore adjusted our *p*-value for significance to 0.017 (0.05 divided by 3). The assumptions for these analyses with regard to homoscedasticity, normality of residuals, and linearity of relationships were met.

Spelling and verbal intelligence were not included in these analyses, as they did not correlate with any of the perceived negative consequences. Word reading fluency was chosen over pseudoword reading fluency because it better reflects reading ability. We could not include both measures in the model because of the strong correlation between word and pseudoword reading. Note that an analysis with pseudoword reading fluency yielded the same results.

The results of the regression analyses are presented in Table 4. The factors that were entered in the models significantly explained variance for all three types of perceived consequences (academic: 25%, anxiety: 28%, depression: 32%). Cohen’s *f*^*2*^ effect sizes were medium (0.326 for the academic domain) to large (0.389 and 0.456 for the academic and depression domains, respectively; Cohen (1988)). Word reading fluency did not significantly contribute to any of the three types of perceived negative consequences. In contrast, self-perceived literacy disability significantly contributed to more perceived consequences in all domains. Higher outcomes on the socio-emotional person factor coping contributed to less depression consequences only. As for the environmental factors, literacy demands did not uniquely contribute to any type of perceived consequences, despite its correlation with all three types of perceived consequences. Although positive correlations had been found between support of the educational institution and perceived consequences, in our regression analysis, better support of the institution did not independently contribute to perceived negative consequences. However, more positive attitudes of lecturers and peers were related to less perceived negative anxiety and depression consequences.

**Table 4:** Standardized beta coefficients and *p*-values from the regression analyses per domain of perceived negative consequences

	Academic		Anxiety		Depression	
Factor	β	*p*	β	*p*	β	*p*
Word reading fluency	.068	.411	− .071	.384	.103	.193
Self-perceived disability	.275	.001	.347	.000	.228	.005
Coping strategies	− .182	.028	− .007	.932	− .197	.013
Literacy demands	.169	.056	.106	.216	.163	.034
Support institution	− .206	.020	− .068	.425	− .145	.085
Attitudes of lecturers and peers	− .089	.317	− .293	.001	− .219	.011
Total R^2^	.246		.280		.313	

A *p*-value lower than .017 denotes a significant effect

## Discussion

Dyslexia is associated with problems in educational attainment as well as feelings of anxiety and depression. Research has generally not focused on the factors that determine the degree to which individuals with dyslexia *perceive* negative consequences from their disorder, i.e., contribute these problems/feelings to the disorder. The ICF model of disorders (WHO, 2001) indicates that experienced negative consequences from a disorder are influenced by person and environmental factors. Based on this ICF model, we investigated whether perceived negative consequences from dyslexia (academic, anxiety, and depression) were related to cognitive person factors (literacy ability and general verbal ability), socio-emotional person factors (self-perceived literacy disability and coping), and environmental factors (literacy demands, support of the educational institution and attitudes of lecturers and peers) among Dutch university students.

We found that mean negative academic consequences and feelings of anxiety attributed to dyslexia (± 3 out of 5) were numerically higher than those of depression (± 2 out of 5). With respect to the relations between these experienced consequences and the factors we investigated, weak correlations were found between the cognitive person factor reading ability and perceived consequences with respect to anxiety and depression, whereas no relations were found with spelling and verbal intelligence. As for the socio-emotional person factors and environmental factors, moderate associations were found of all of these factors with all three types of perceived consequences.

Subsequent regression analyses indicated that a moderate percentage of variance in the perceived negative consequences could be explained by the factors included in our study (25% in the academic domain and 28% and 32% for feelings of anxiety and depression, respectively). These analyses also provided insight in the unique contributions of the factors to the different types of perceived consequences. We found that the three types of perceived consequences were related to different sets of person and environmental factors. The cognitive person factors (literacy ability and general verbal ability) did not provide unique contributions to any of the perceived consequences. This was also the case for the environmental factors literacy demands and support of the institution, despite their moderate correlations with all three types of perceived consequences. With respect to socio-emotional factors, self-perceived literacy disability contributed to all perceived consequences, and coping contributed to perceived depression consequences. Regarding environmental factors, attitudes of teachers and peers contributed to perceived anxiety and depression consequences. In sum, cognitive person factors, particularly the severity of the reading disorder, did not play a role in the perceived consequences for students with dyslexia, but socio-emotional person and environmental factors did.

### Perceived consequences and cognitive person factors

On the basis of the ICF model (WHO, 2001) and an earlier study by Elbro (2010), we expected that the cognitive person factors (literacy ability and general verbal ability) would influence the extent to which negative consequences are attributed to dyslexia. We did not, however, find a relation. Possibly, this is due to the homogeneity of our participant sample, with a subsequent restriction of range in performance. We included only students with a formal dyslexia diagnosis and as a result performance on reading and spelling ability was generally low (in line with Tops et al., 2012). Furthermore, all the participating students were enrolled in university or university of applied science programs. The fact that all the students in our sample had entered a high level of education might in itself be a reason for the absence of a contribution of general verbal ability to perceived negative academic consequences. If general verbal abilities lead to the pursuit of high academic goals, the failure to achieve this goal due to literacy problems might increase the perceived academic consequences. However, this might not be the case in our sample.

The earlier study among a less homogenous group by Elbro (2010) did find that lower literacy skills were related to more perceived academic consequences of dyslexia, with an additional effect of higher general verbal ability (vocabulary). In Elbro’s study, the sample included “adults with variable but generally poor reading (and spelling) abilities” (p.5), who were recruited via reading courses and education forms with a high percentage of poor readers. His sample therefore probably included a broader range of readers who, as a result, showed more variation in literacy performance than ours. With respect to educational attainment, his sample also showed more variability. It included participants with a broader range of educational outcomes, of whom most had not finished vocational training, and only a few had finished any further education. As such, some of these participants with high(er) general verbal skills might have attributed their low educational outcomes to their literacy problems.

An additional explanation for the finding that there was no contribution of literacy abilities to perceived consequences in the current study may reside in the type of tasks we used to examine literacy performance. We included word-level list reading and spelling measures used in the process of diagnosing dyslexia. This is in line with the assumption that dyslexia is a severe and persistent disorder in word-level literacy reflected in the dyslexia diagnosis (DSM-5, American Psychiatric Association, 2013). Yet, in the advanced stage and level of education of the students, literacy demands exceed the word level as text reading and essay writing are required (Bazen et al., 2020; Moojen et al., 2020). It would therefore be worthwhile to evaluate both word-level literacy, the core characteristics of dyslexia (DSM-5 American Psychiatric Association, 2013; Tijms et al., 2021), and more demanding literacy abilities (text reading fluency, reading comprehension) in future research.

### Perceived consequences and socio-emotional person factors

Contrary to actual literacy ability, lower self-perceived literacy disability did result in attributing more negative consequences to dyslexia in all three domains. Whereas actual literacy ability was limited to word list reading and single word spelling, the questions within the self-perceived literacy scale included those that tapped into broader literacy skills (e.g., “I have difficulties with reading the required literature for my education”). As a result, this scale may have been more representative for literacy difficulties students encounter at university, and therefore more strongly related to perceived consequences.

The finding that the subjective evaluation of literacy problems was related to perceived consequences of dyslexia, whereas the objective measures of literacy were not, could also mean that self-perceived literacy ability is influenced by more than competence alone (Fives et al., 2014; Marsh & Craven, 2006). Indeed, in a study by Frederickson and Jacobs (2001), children with a dyslexia diagnosis judged their literacy competence to be lower than their peers without a dyslexia diagnosis, but with the same actual level of literacy ability. Also, qualitative studies indicate that parts of self-concept such as literacy ability can be influenced by factors earlier in education, for instance by support within the school environment and the quality of early tutoring by teachers and peers (Humphrey, 2003; Humphrey & Mullins, 2002). Self-perceived literacy ability itself is thus susceptible to person and environmental factors, just as perceived consequences of dyslexia are. Because of the cross-sectional design of the current study, we cannot determine the developmental relationship between self-perceived and objective literacy abilities.

With respect to the other socio-emotional factors, there was no unique contribution of coping to perceived academic consequences despite a correlation between these factors as well as previous findings regarding the relation between coping and academic success (Alexander-Passe, 2006; Singer, 2008). Our study also showed that coping contributed to perceived depression consequences while it was not related to perceived anxiety consequences, even though in previous studies a relation was found between coping and both anxiety (Carroll & Iles, 2006) and depression (Alexander-Passe, 2006) in individuals with dyslexia. Our assessment of coping targeted mainly problem-focused coping, whereas we did not measure emotion-based coping. This could possibly account for the absence of contribution of coping to perceived anxiety consequences. However, an independent contribution of coping to perceived depression consequences was found. Perhaps, positively responding to the items in this scale required a certain degree of self-confidence (Armstrong & Humphrey, 2009; Haft et al., 2016; Singer, 2008). For example, the item “I know how to deal with my dyslexia” may tap into a degree of self-esteem that also helps to decrease the feelings of depression or the inclination to attribute feelings of depression to dyslexia (McArthur et al., 2016, 2022). The influence of coping on perceived depression consequences of dyslexia might at least in part reflect the confidence of participants rather than the ability of coping itself.

### Perceived consequences and environmental factors

Although the environmental factor literacy demands correlated with perceived consequences attributed to dyslexia in all three domains, it did not uniquely contribute to any of them. This indicates that the relation between literacy demands and perceived consequences was not independent of other factors. We suspect that self-perceived literacy ability and literacy demands overlapped in such a manner that self-perceived literacy ability accounted for the variance in literacy demands. Self-perceived literacy ability then presumably contributed more strongly to the types of perceived consequences than literacy demands alone, because it captured a broader set of skills. This overlap resulted in a unique contribution of self-perceived literacy disability and not of literacy demands to perceived consequences.

With respect to support of the educational institution as well as attitudes of lecturers and peers, qualitative studies reported that both were related to actual academic consequences of dyslexia as well as emotional problems (Gibson, 2012; Nelson & Gregg, 2012). Although moderate correlations were found between both factors and all three types of perceived consequences, only attitudes of lecturers and peers uniquely contributed to perceived anxiety and depression consequences. These findings stress the important role of students and lecturers in reducing the perception of depression and anxiety consequences among students with dyslexia in an academic setting.

In line with the ICF model (WHO, 2001), our results showed the relevance of socio-emotional person and environmental factors for the perceived consequences from dyslexia. Furthermore, the factors that determine the perceived consequences differ per domain (academic, anxiety, and depression). Our findings complement recent studies towards the influence of person and environmental factors on the manifestation of dyslexia (e.g., Catts & Petscher, 2022) and secondary consequences such as anxiety and depression. While these previous studies and meta-analyses (Francis et al., 2019; McArthur et al., 2020, 2022) showed direct relations between person and environmental factors and the consequences of dyslexia, our study showed that the *perception* of negative consequences, directly attributed to dyslexia, is influenced by such factors as well.

#### Limitations and future directions

Although our study provides more insight into the factors that are related to perceived negative consequences from dyslexia, it is qualified by some limitations. One limitation concerns the selection of the person and environmental factors, which was not exhaustive. An important environmental factor that we did not include, for instance, was the attitudes and support of people in the personal environment of the student. While in the current study we chose to focus on support within the academic environment directly, research has shown the important role of family and friends in handling academic and emotional difficulties during education as a result of reading problems in children (Idan & Margalit, 2014; Singer, 2008; Al-Yagon, 2016) as well as adults (Nalavany et al., 2011). Although the role of family and friends for university students is not exactly known, we believe that including a factor that measures support outside of the academic environment in a future study may contribute to further understanding the role of the environment in attributing consequences to dyslexia.

In addition, studies have shown that problem-focused coping strategies (e.g., a stepwise approach in solving problems) tend to be more effective than emotion-based coping strategies (e.g., denial or looking for emotional support), although within these types of strategies there are approaches that are more and less effective depending on the individual (e.g., Baker & Berenbaum, 2007). By differentiating between types of coping as predicting factors in a future study, more specific information could be provided about the relation between types of coping and experienced negative consequences attributed to dyslexia than the current study offers. This would especially be the case within a longitudinal design, as this would offer the opportunity the look into the effect of (changing) coping strategies with time, and/or the influence of treatment and the moment of a dyslexia diagnosis on coping strategies.

Furthermore, the current study investigated experienced consequences attributed to dyslexia in the academic domain and the domain of mental health. We did not assess whether the participants displayed actual difficulties regarding academic outcomes, depression, and anxiety. Meta-analyses (Francis et al, 2019; Gibby-Leversuch et al., 2019; McArthur et al., 2020) and analyses on longitudinal data (McArthur et al., 2022) have shown the existence of measurable problems in these areas for people with dyslexia. It is therefore likely, but not established in our study, that such difficulties would also be (partly) present in our sample of participants. By including measures of both actual and perceived consequences, it can be investigated to what extent negative perceived consequences are determined by the actual presence of academic problems and feelings of anxiety and depression.

The current study focused on students in higher education. Possibly, the perception of negative consequences of dyslexia and related factors may be susceptible to the stage of education. A longitudinal design could shed light on this issue. Furthermore, our cross-sectional study cannot state causal (developmental) relations in a way that longitudinal studies into perceived consequences can. Such a longitudinal design can actually disentangle causes and (perceived) consequences of dyslexia (e.g., McArthur et al., 2022). A future study with a longitudinal design would also help gain insight in the interplay between person and environmental factors and perceived consequences, as both perception of consequences and influencing factors may change over time. Self-perceived literacy disability can change throughout development (Stone & May, 2002), and the perception of consequences itself is likely to be influenced by, for example, support early in education (Humphrey, 2003).

Within such a longitudinal design, it would be especially insightful to include information regarding the dyslexia diagnosis. Research has shown that receiving a dyslexia diagnosis can lead to both positive feelings of understanding and negative feelings due to, for example, stigmatization (Alexander-Passe, 2015; Daley & Rappolt-Schlichtmann, 2018; Sims et al., 2021). Additionally, these feelings can change with time (Battistutta et al., 2018; Pino & Mortari, 2014). A qualitative study by Gibby-Leversuch et al. (2021) showed that a dyslexia label is related to more positive perceptions in the environment. More specifically, this study showed that more understanding and support from the environment are experienced by individuals with a dyslexia label than by those without such a label but with comparable literacy difficulties. Incorporating information regarding the dyslexia label of individuals in longitudinal research could be informative with respect to the influence of these changing perspectives from both the individual and the environment on the perceived consequences of dyslexia.

With respect to treatment that follows the diagnosis itself, an influence on perceived consequences as well as on other predicting person and environmental factors can be anticipated. Both the success of literacy training itself and psycho-education aimed at accommodating towards learning disabilities may establish such influences, because of their interplay with, for example, coping and self-perceived ability. Also, the perception of (negative) consequences of dyslexia itself may be targeted in such training. In addition, the notion of perceived consequences may be incorporated in future studies towards the effects of treatment success in order to build on scientific and (psycho-educational) treatment knowledge in this domain. In line with this, it would be an interesting direction for future research to investigate the perceived efficacy of interventions and/or treatment in relation to perceived consequences, as these may change when, for example, accommodations are improved. This could be addressed in an intervention study in which measures towards perceived negative consequences as well as perception of efficacy of interventions are included.

## Conclusion

The current study shows that the amount of perceived negative consequences that university students experience due to their dyslexia can partly be accounted for by socio-emotional and environmental factors. Our findings are in line with the ICF model of disorders (WHO, 2001), as it was shown that the experienced consequences of dyslexia are susceptible to factors outside of the disorder itself. As such, our findings contribute to a growing body of research aimed at understanding the factors that influence the perceived consequences of dyslexia (e.g., McArthur et al., 2020; Haft et al., 2016). Given that these perceived consequences cannot be fully accounted for by objective reading and writing problems, the influence of socio-emotional and environmental factors should be taken into account in further research in this area. Given our findings, we believe that attention towards both experienced negative consequences as well as person and environmental factors that are of influence on this perception should be included in the process of diagnosing and supporting individuals with dyslexia.

## Supplementary Information

Below is the link to the electronic supplementary material.

Supplementary file1 (DOCX 21 KB)
