# Supplementary material for: Perceived negative consequences of dyslexia: the influence of person and environmental factors
Source: Ann Dyslexia. 2022 Nov 30;73(2):214–34. doi: 10.1007/s11881-022-00274-0 (PMC10247831; doi:10.1007/s11881-022-00274-0)
Supplement: Supplementary file 1 — Supplementary file1 (DOCX 21 KB) [file 11881_2022_274_MOESM1_ESM.docx]

**Supplementary Material**

**Questionnaire Perceived Negative Consequences and Socio-emotional and Environmental Factors^[[1]](#footnote-1)^

 Perceived negative academic consequences (8 items, five-point Likert scale)**

If I did not have dyslexia, I would get better grades.

If I did not have dyslexia, I would need less time to study.

If I did not have dyslexia, I would write better papers.

If I did not have dyslexia, I would better understand the required literature.

If I did not have dyslexia, I would graduate sooner.

If I did not have dyslexia, I would be able to take more subjects at once.

If I did not have dyslexia, I would be better at planning for my education.

If I did not have dyslexia, I would be better at giving presentations.

**Perceived negative anxiety consequences (5 items, five-point Likert scale)**

Due to my dyslexia I do not dare to send emails, because I am afraid I made spelling

mistakes.

Due to my dyslexia I am nervous for exams that require a lot of writing.

Due to my dyslexia I am nervous for exams that require a lot of reading.

Due to my dyslexia I am afraid there are spelling mistakes on my slides when I have to give a presentation.

Due to my dyslexia I feel annoyed.

**Perceived negative depression consequences (9 items, five-point Likert scale)**

Due to my dyslexia I feel unworthy.

Due to my dyslexia I feel down.

Due to my dyslexia I feel sad.

Due to my dyslexia I feel like a failure.

Due to my dyslexia I feel despondent about my future.

Due to my dyslexia I feel worn out.

Due to my dyslexia I am worried about finding a job after studying.

Due to my dyslexia I doubt myself.

Due to my dyslexia I am less confident that things will succeed.

**Self-perceived literacy disability (9 items, five-point Likert scale)**

I have a slower reading speed than my peers.

I have difficulties with reading the required literature for my education.

I have difficulties with reading words I have never seen before (such as town names).

I have difficulties with writing papers.

I have difficulties with spelling words such as pinguïns, relaxed and cabaretier.

I have difficulties with structuring my written texts.

I have difficulties with spelling in foreign languages.

I have difficulties with reading English words.

I am a … reader (very slow – very fast)

**Coping strategies (5 items, five-point Likert scale)**

I know how to deal with my dyslexia.

I have the appropriate reading strategies to make the reading of long texts easier.

I have come up with tricks to better understand educational literature.
I have several learning strategies
I make use of mnemonics/memory tricks with spelling.

**Literacy demands (6 items, five-point Likert scale)**

Exams contain a lot of text.

Exams require a lot of writing.

Spelling mistakes on exams are strictly assessed.

Spelling mistakes in papers are strictly assessed.

I have to write a lot of papers for a subject.

I have to read a lot for a subject.

**Support from the educational institution (3 items, five-point Likert scale)**

I am aware of the accommodations my educational institution offers for dyslexic students.

I am satisfied with the accommodations my education institution offers for dyslexic students.

I get the appropriate type of support from my university.

**Attitudes of lecturers and peers (7 items, five-point Likert scale)**

Lecturers are understanding of my reading and spelling difficulties.

Lecturers take the time if I need more support due to my dyslexia.

I receive unpleasant remarks from lecturers if I have made a spelling mistake.

Peers are understanding if I make reading or spelling mistakes.

I receive unpleasant remarks from peers if I have made a spelling mistake.

Lecturers think it is annoying if I have requested accommodations.

Lecturers call into question if I need extra accommodations.

1. Items included after principle component analysis [↑](#footnote-ref-1)
